# Endothelial *MLKL* Inhibition Reduces Hyperoxia‐Induced Bronchopulmonary Dysplasia in Neonatal Mice

**DOI:** 10.1111/jcmm.71035

**Published:** 2026-01-29

**Authors:** Junjie Ning, Junchao Deng, Yating Sang, Lina Qiao

**Affiliations:** ^1^ Department of Pediatrics, West China Second University Hospital Sichuan University Chengdu China; ^2^ Key Laboratory of Birth Defects and Related Diseases of Women and Children, Ministry of Education Sichuan University Chengdu China; ^3^ Department of Pediatrics, Sichuan Provincial People's Hospital University of Electronic Science and Technology of China Chengdu China; ^4^ NHC Key Laboratory of Chronobiology Sichuan University Chengdu China

**Keywords:** bronchopulmonary dysplasia, endothelial cell, hyperoxia, *MLKL*, necroptosis

## Abstract

Bronchopulmonary dysplasia (BPD) remains a severe complication in premature infants requiring prolonged oxygen therapy, with vascular endothelial dysfunction recognised as a critical contributor to disease progression. Mixed lineage kinase domain‐like protein (*MLKL*)‐mediated necroptosis, an essential form of regulated cell death implicated in various pulmonary disorders, has not been fully investigated in the context of BPD. Here, we utilised a neonatal mouse model of hyperoxia exposure to elucidate the role and mechanisms of *MLKL*‐mediated necroptosis in BPD pathogenesis. Our analysis demonstrated morphological characteristics of necroptosis in pulmonary vascular endothelial cells (ECs) under hyperoxic conditions, accompanied by significant elevation of *MLKL* protein levels and marked upregulation of *MLKL* gene expression specifically in vascular ECs. Administration of the *MLKL* inhibitor necrosulfonamide (NSA), either immediately postnatally or at postnatal day 7, effectively mitigated lung injury, preserved alveolar structure and partially restored pulmonary vascular growth. Moreover, *MLKL* conditional knockout in ECs significantly attenuated both structural and functional pulmonary abnormalities induced by hyperoxia. Collectively, our findings indicate that *MLKL*‐mediated necroptosis in vascular ECs plays a pivotal role in hyperoxia‐induced BPD. Therapeutically targeting *MLKL* to maintain endothelial integrity presents a promising approach to prevent or alleviate BPD in premature infants.

**Abbreviations**
ALI: acute lung injury
BPD: bronchopulmonary dysplasia
CKO: conditional knockout
ECs: endothelial cells
EF50: mid‐expiratory flow rate
H&E: haematoxylin and eosin
KEGG: Kyoto Encyclopedia of Genes and Genomes
MAA: mean alveolar area
MLI: mean linear intercept
*MLKL*
: mixed lineage kinase domain‐like protein
NSA: necrosulfonamide
RAC: radial alveolar count
*RIPK1*
: receptor‐interacting protein kinase 1
*RIPK3*
: receptor‐interacting protein kinase 3
RT–qPCR: quantitative real‐time PCR
TEM: transmission electron microscopy
TV: tidal volume
VEGF: vascular endothelial growth factor

## 1 Introduction

Northway first described bronchopulmonary dysplasia (BPD) in 1967 as a chronic pulmonary disorder occurring in premature infants who required prolonged mechanical ventilation [1]. Since then, BPD has come to be recognised as a complex, multifactorial and multistage disease that is closely associated with increased mortality, long‐term respiratory morbidity, neurodevelopmental impairment and substantial healthcare burden [2, 3]. Prolonged exposure to hyperoxia is considered a major contributing factor in its pathogenesis [4, 5, 6]. Over the past two decades, advances in neonatal care, including the widespread use of antenatal glucocorticoids, exogenous surfactants and protective ventilation strategies, have markedly altered both the epidemiology and pathological features of BPD. Consequently, the definition of BPD has shifted from a fibrocystic lung disease primarily observed in late preterm infants to a disorder predominantly affecting extremely preterm infants born before 29 weeks of gestation, characterised by pulmonary parenchymal dysplasia and dysregulated vascular development [7, 8, 9].

Recent studies have shown that the pulmonary vasculature plays not only a crucial role in alveolar formation but also a central role in maintaining alveolar structural stability and function throughout life [10]. Appuhn SV reported that disruption of the alveolar capillary network often occurs prior to injury of alveolar epithelial cells [11]. In addition, abnormal alveolar microvasculature has also been identified in lung tissues from infants who died of BPD [12]. These findings suggest that BPD is not merely an airway disease but may also involve disordered pulmonary angiogenesis, which likely plays a key role in its pathogenesis. Early studies further indicated that pulmonary vascular endothelial cells (ECs) are primary targets of hyperoxic stress [13, 14], and endothelial dysfunction in premature infants is considered an important contributing factor to the development of BPD [15, 16]. Collectively, these observations highlight that in‐depth investigation of pulmonary vascular abnormalities in BPD, particularly the regulatory mechanisms of ECs, is essential for a comprehensive understanding of the disease process.

Necroptosis is a regulated form of cell death mediated by receptor‐interacting serine/threonine protein kinase 1 (*RIPK1*), *RIPK3* and the terminal effector *MLKL* [17, 18]. Upon activation, *MLKL* oligomerises and translocates to the plasma membrane, causing membrane disruption and the release of large quantities of damage‐associated molecular patterns, including high mobility group box 1, mitochondrial DNA and ATP, thereby exacerbating inflammation [19]. Notably, *RIPK1* and *RIPK3* are involved in multiple signalling pathways beyond necroptosis, including apoptosis and inflammatory signalling [20, 21]. By contrast, as the terminal effector of necroptosis, *MLKL* directly mediates membrane disruption and cell lysis, making it a more specific determinant of necroptotic cell death [22].

From a mechanistic perspective, multiple forms of regulated cell death, including apoptosis and autophagy‐dependent cell death, are increasingly recognised as central drivers of BPD [23, 24]. However, direct evidence supporting a role for necroptosis in BPD is still limited. Notably, hyperoxia has been shown to directly activate the necroptosis pathway, leading to substantial lung cell death [25]. Hyperoxia‐induced acute lung injury is associated with increased necrotic cell death and elevated expression of *RIPK1*, *RIPK3* and *MLKL*, whereas pretreatment with free radical scavengers attenuates oxidative stress, necrosis and the expression of these key necroptotic mediators, thereby alleviating lung pathology [6]. Moreover, activation of necroptosis not only exacerbates cell death but also amplifies pulmonary inflammation through the release of inflammatory mediators [19], a process that may in turn contribute to impaired alveolar development and BPD‐like pathological changes [26]. Collectively, these findings suggest that the *RIPK1*/*RIPK3*/*MLKL* signalling axis may contribute to the initiation and progression of BPD, although the precise molecular mechanisms remain incompletely understood.

Given the vulnerability of pulmonary ECs to hyperoxia‐induced injury and the critical role of vascular development in alveolarisation, this study aimed to determine the role of *MLKL*‐mediated necroptosis in pulmonary vascular ECs in the pathogenesis of BPD under hyperoxic conditions.

## 2 Materials and Methods

### 2.1 Animal Model

C57BL/6J specific pathogen‐free mice were obtained from Jiangsu Jicui Yaokang Biotechnology Co. Ltd. Floxed *MLKL* (*MLKL* ^loxp/loxp^) mice and EC–specific VE‐cadherin (PAC)‐CreERT2 mice were kindly provided by Dr. Nan Yang (Sichuan University).

For hyperoxia exposure, neonatal mice were randomly assigned at birth (P0) to either a normoxia group (21% O_2_) or a hyperoxia group (60% O_2_), with 12 pups per group. Hyperoxia‐exposed mice were housed in a sealed Plexiglas chamber maintained at 22°C–26°C, 50%–60% humidity and CO_2_ < 0.5% for 14 days. Normoxic control mice were maintained in room air under identical conditions.

To block *MLKL*‐mediated necroptosis in vivo, C57BL/6J neonatal mice were divided into three groups: air control, necrosulfonamide (NSA) T_0th_ and NSA T_7th_ groups, with six mice per group. NSA is a selective inhibitor of necroptosis that prevents MLKL oligomerisation and translocation to the plasma membrane, thereby blocking its execution function [27, 28]. The air control group was maintained in room air (21% O_2_) for 14 days. The NSA T_0th_ group was continuously exposed to hyperoxia (60% O_2_) from birth and received daily subcutaneous injections of NSA (1.6 mg/kg/day) from P0 to P14. The NSA T_7th_ group was also exposed to hyperoxia from P0 to P14, with NSA administration (1.6 mg/kg/day, subcutaneous) initiated at P7 and continued until P14.

VECad‐Cre(+/−) *MLKL* ^loxp/loxp^ mice were generated by cross‐breeding *MLKL* ^loxp/loxp^ mice with VE‐cadherin (PAC)‐CreERT2 mice. VECad‐Cre(+/−) *MLKL* ^loxp/loxp^ mice were subcutaneously injected with tamoxifen (20 mg/kg/day) for five consecutive days after birth to induce *MLKL* conditional knockout in ECs (*MLKL* EC‐CKO; *MLKL* ^iΔEC/iΔEC^). Neonatal mice were assigned to three experimental groups: (1) *MLKL* ^loxp/loxp^ mice maintained under normoxic conditions (21% O_2_); (2) *MLKL* ^loxp/loxp^ mice exposed to hyperoxia (60% O_2_) from birth for 14 days and (3) *MLKL* ^iΔEC/iΔEC^ mice exposed to hyperoxia (60% O_2_) from birth for 14 days. Each group included three mice.

### 2.2 Histopathological Analysis

Lung samples were embedded in paraffin, heated, dewaxed and hydrated with xylene. These sections were then stained with haematoxylin and eosin (H&E) and Masson's trichrome to evaluate histopathological alterations and collagen deposition. CD31 immunohistochemical staining was performed to detect vascular ECs. The alveolar mean linear intercept (MLI), radial alveolar count (RAC) and mean alveolar area (MAA) were measured following previously established protocols [29, 30, 31].

### 2.3 Respiratory Metrics

Mouse pulmonary function was evaluated using a small animal noninvasive pulmonary function test system (WBP, DSI Buxco). The respiratory parameters were calculated by the software provided by the manufacturer. After the measurement data stabilised, the breathing data of each mouse were continuously recorded for 5 min, and the mean value was calculated as an indicator of pulmonary function.

### 2.4 RNA Isolation, Reverse Transcription and Quantitative Real‐Time PCR (RT–qPCR)

Total RNA was extracted from lung ECs using TRIzol reagent according to the manufacturer's instructions. RNA concentration and purity were assessed spectrophotometrically, and RNA integrity was verified prior to downstream analysis. One microgram of total RNA was reverse‐transcribed into cDNA in a 20 μL reaction mixture using the PrimeScript RT reagent Kit (Takara, Japan). RT–qPCR was performed using TB Green Premix Ex Taq II (Tli RNaseH Plus; Takara, Japan) on a QuantStudio 3 Real‐Time PCR System (Thermo Fisher Scientific, USA). Each sample was analysed in triplicate. β‐actin was used as the internal reference gene after confirming its stable expression across experimental groups. Relative gene expression levels were calculated using the 2^−ΔΔCt^ method. Primer specificity was confirmed by melting curve analysis. The primer sequences were as follows: *MLKL* (forward: agcaagaagtcccatatttgga; reverse: gctgacatctgaaacggtattc) and β‐actin (forward: ctacctcatgaagatcctgacc; reverse: cacagcttctctttgatgtcac).

### 2.5 Western Blot

Total proteins were extracted from ECs using RIPA lysis buffer. The proteins were separated via SDS–PAGE and then transferred to nitrocellulose filter membranes. After blocking with 5% skim milk, the membranes were incubated overnight at 4°C with anti‐*MLKL* (Abcam, ab243142, 1:2000) and anti‐β‐actin (ABclonal, AC026, 1:50000) primary antibodies. Afterwards, the membranes were exposed to an HRP‐conjugated secondary antibody for 2 h at room temperature. Enhanced chemiluminescence reagents were used to visualise the bands.

### 2.6 Transmission Electron Microscopy (TEM)

Lung tissue samples were initially fixed in 3% glutaraldehyde followed by 1% osmium tetroxide, dehydrated using a graded acetone series and embedded in Ep812. Ultrathin sections were mounted onto copper grids and sequentially stained with uranyl acetate and lead citrate at room temperature. The ultramicroscopic structure of the cells was observed via TEM (JEM‐1400FLASH, Japan), and a specific area was selected to observe pathological characteristics in detail.

### 2.7 Immunofluorescence

Lung tissues were fixed, dehydrated, embedded and sectioned. Sections were deparaffinised, subjected to antigen retrieval in citrate buffer and blocked with 10% serum for 30 min. They were then incubated overnight at 4°C with primary antibodies against *MLKL* (Affinity, DF7412, 1:500) and RIP3 (Cell Signalling, 95702S, 1:1000). Following PBS washes, sections were incubated with a fluorescent secondary antibody and counterstained with 4′,6‐diamidino‐2‐phenylindole (DAPI). Finally, the sections were mounted in antifade medium and imaged using a Carl Zeiss LSM880 laser scanning confocal microscope (Prenzlauer, Berlin, Germany).

### 2.8 Single‐Cell RNA Sequencing (scRNA‐Seq)

Lungs were perfused with PBS, minced and digested at 37°C for 35 min in an enzyme mixture containing Dispase II (200 μg/mL), Collagenase I (200 μg/mL) and DNase I (40 μg/mL). After digestion, the samples were filtered, and the red blood cells were lysed. The resulting single cells were resuspended in MACS buffer (DPBS supplemented with 0.1% BSA and 2 mM EDTA), ensuring cell viability exceeded 80%. Approximately 10,000 cells were then loaded into the 10X Genomics Chromium system following the manufacturer's instructions for the Chromium Single Cell 3′ Kit (V3). Additionally, gel bead emulsions (GEMs) containing barcoded beads were generated via microfluidics, followed by reverse transcription, cDNA amplification and library construction. Libraries were sequenced on an Illumina NovaSeq 6000 platform with 150 bp paired‐end reads. The sequencing data were converted to FASTQ format using bcl2fastq, aligned to the mm10‐1.2.0 reference transcriptome using Cell Ranger (v6.1.1) and subjected to dimensionality reduction, clustering and downstream analyses with Seurat (v4.0). Each sample group was composed of pooled lung tissues from three BPD and AIR model mice, confirmed by pathological examination.

### 2.9 Statistical Analysis

All data were analysed using GraphPad Prism 9.0 software or R. The Shapiro–Wilk test was first employed to determine whether the data followed a normal distribution. The independent samples *t* test was utilised for normality assumptions and the Mann–Whitney *U* test for non‐normally distributed data to conduct statistical comparisons. Statistical significance was set at *p* < 0.05, with the following notation for significant differences: * *p* < 0.05, ** *p* < 0.01, *** *p* < 0.001, **** *p* < 0.0001.

## 3 Results

### 3.1 Hyperoxia Disrupted Alveolar and Vascular Growth and Impaired Respiratory Function in Neonatal Mice

**FIGURE 1:**
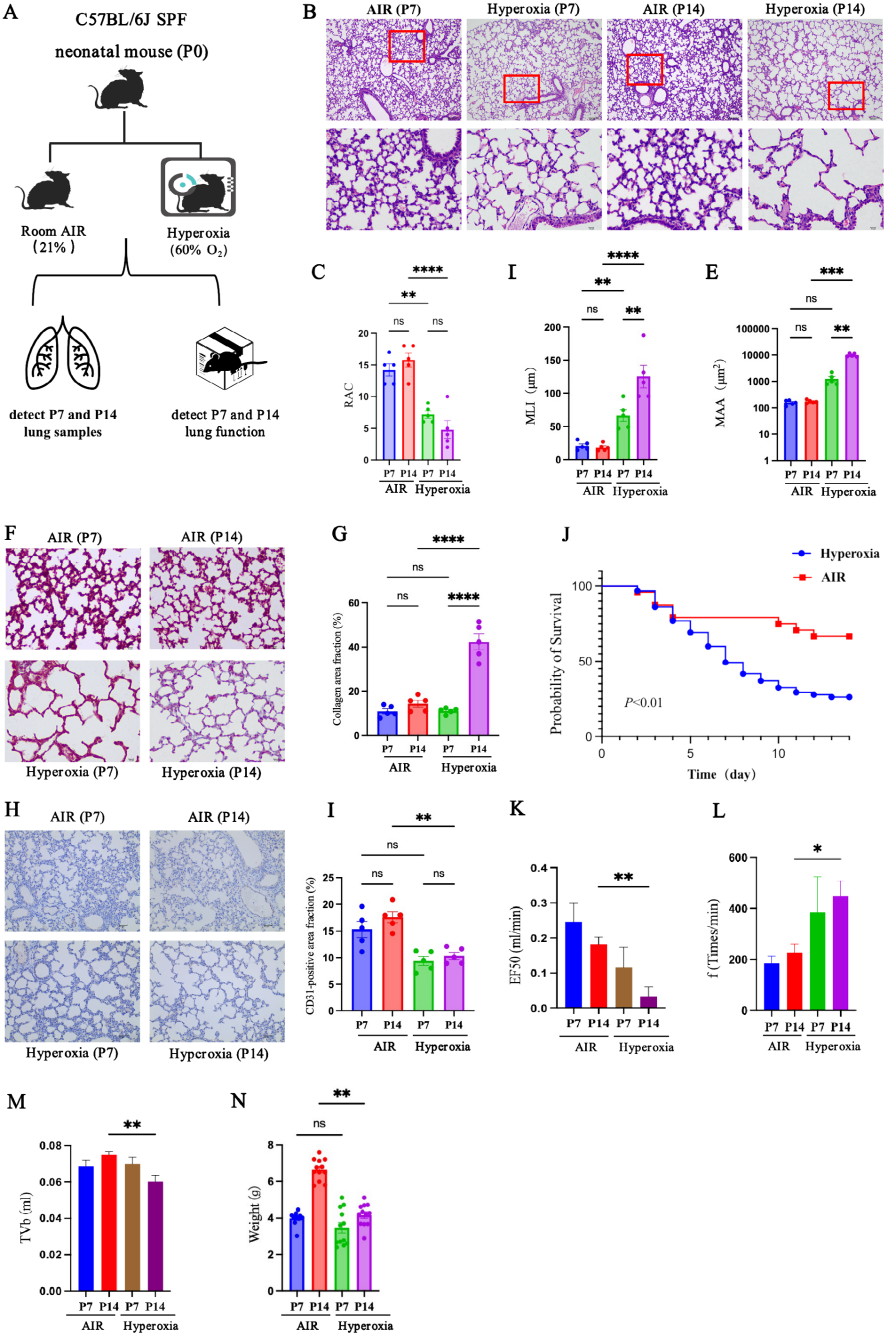
Effects of hyperoxia exposure on alveolar and vascular growth and respiratory function in neonatal mice. (A) Neonatal C57BL/6 mice were raised under 21% O_2_ or 60% O_2_ conditions from birth until P7 or P14. At these time points, respiratory function was assessed, and lung tissues were examined; (B) Representative H&E staining images. The top panels show low‐magnification images (scale bar = 100 μm), and the bottom panels show high‐magnification images (scale bar = 10 μm); (C–E) Quantification of the RAC (C), MLI (D) and MAA (E) in the images in (B); (F) Representative Masson staining images (scale bar = 10 μm); (G) Quantification of the data in (F); (H) Representative CD31 immunohistochemical staining images (scale bar = 40 μm); (I) Quantification of the data in (H); (J) Survival curve analysis; (K–M) Analysis of respiratory function; (N) Analysis of body weights. * *p* < 0.05, **p < 0.01; ***p < 0.001; ****p < 0.0001; ns > 0.05.

In this study, we evaluated lung tissue changes in C57BL/6J neonatal mice exposed to hyperoxia (60% O_2_) or room air (21% O_2_) for 7 or 14 days (Figure 1A). H&E staining showed that hyperoxia reduced RAC while increasing both MLI and MAA at 7 and 14 days (Figure 1B–E). Masson staining revealed no difference in fibrous area at day 7, but a marked increase by day 14 in the hyperoxia group (Figure 1F,G). Similarly, the CD31‐positive area was comparable on day 7 but significantly lower at day 14 under hyperoxia (Figure 1H,I). Survival analysis demonstrated a reduced survival rate in hyperoxia‐exposed mice (Figure 1J). At day 14, pulmonary function tests indicated an elevated respiratory rate (f), with tidal volume (TV) and mid‐expiratory flow (EF50) significantly decreased (Figure 1K–M). Although body weights were similar at day 7, the hyperoxia group weighed considerably less by day 14 (Figure 1N). These results confirm that 14 days of hyperoxia induce alveolar simplification, fibrosis and impaired microvascular development, along with diminished growth and respiratory function, effectively recapitulating key features of BPD.

### 3.2 Hyperoxia Increased *MLKL* Gene Expression in ECs and Promoted the Development of BPD in Neonatal Mice

**FIGURE 2:**
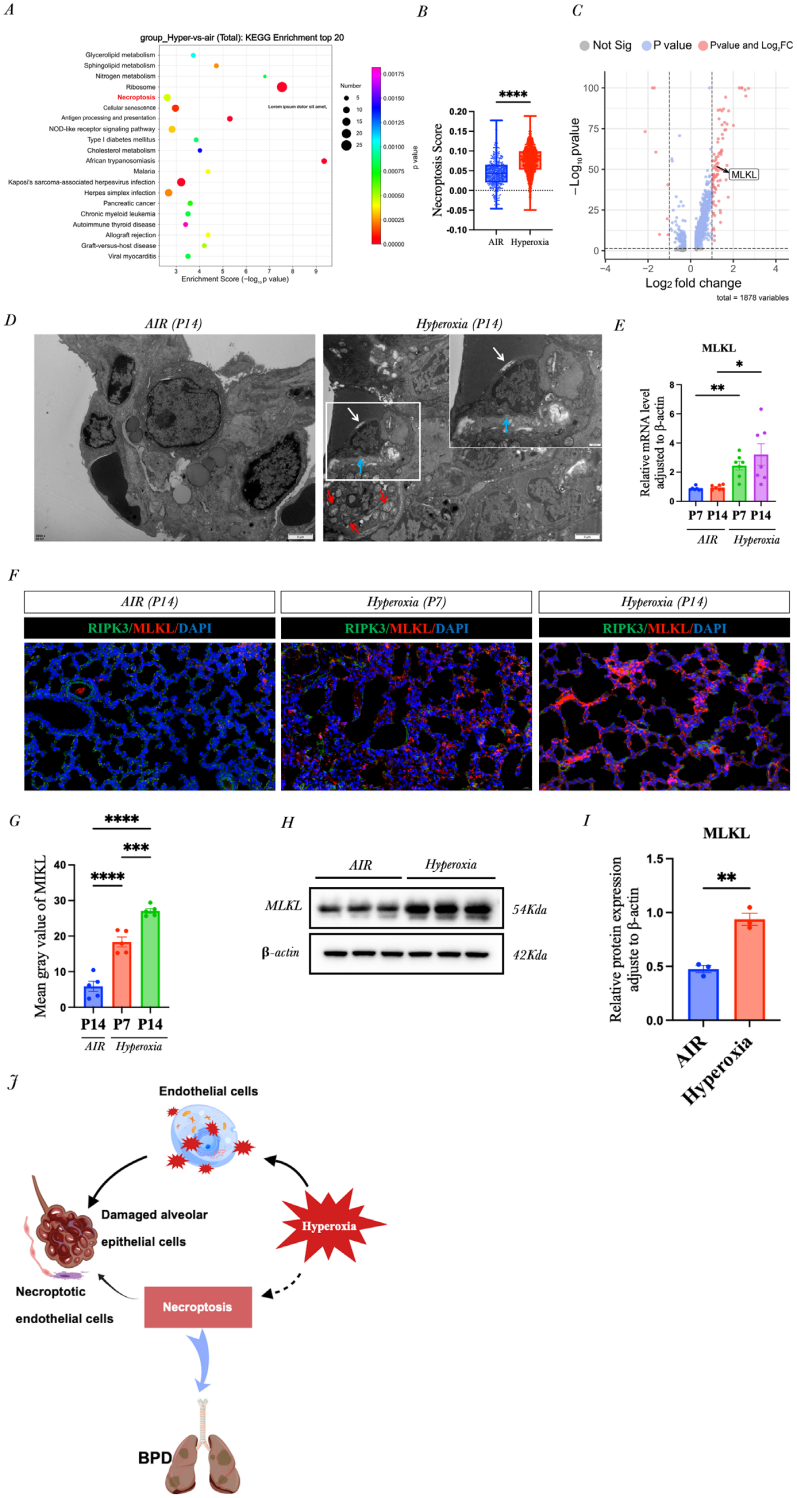
Hyperoxia induced an increase in *MLKL* gene expression in ECs of neonatal mice, which promoted the occurrence and development of BPD. (A) KEGG analysis of the genes in the ECs in the normoxia and hyperoxia groups; (B) Principal component analysis of the endothelial necroptosis scores; (C) Volcano plots of the differentially expressed genes in ECs. *p*‐value < 0.05, |log2FoldChange| ≥ 1; (D) Representative TEM images. The scale bar for the two larger panels is 2 μm, and the scale bar for the small panel is 500 nm; (E) RT–qPCR analysis of *MLKL* mRNA expression in ECs; (F) Representative immunofluorescence labelling images of lung tissues (scale bar = 20 μm); (G) Quantification of the data in (F); (H) Western blot analysis of *MLKL* and p‐*MLKL* in ECs; (I–J) Quantification of the data in (H); (K) Schematic illustration demonstrating that under hyperoxic conditions, increased oxidative stress triggers *MLKL*‐dependent necroptosis in pulmonary cells, particularly in vascular endothelial cells, leading to alveolar simplification and airway remodelling similar to that observed in BPD. *p < 0.05, **p < 0.01; ***p < 0.001; ns > 0.05.

We next evaluated cellular and molecular changes associated with hyperoxia‐induced BPD. Kyoto Encyclopedia of Genes and Genomes (KEGG) analysis of lung ECs at postnatal day 14 revealed significant enrichment of the necroptosis pathway, with an elevated endothelial necroptosis score in the hyperoxia group (Figure 2A,B). Single‐cell RNA sequencing identified 386 differentially expressed genes (361 upregulated and 25 downregulated), among which *MLKL* and *RIPK3* expression were significantly increased in the hyperoxia group (Figure 2C and Figure S1A). Transmission electron microscopy showed that, after 14 days of hyperoxia, type I/II alveolar epithelial cells exhibited mitochondrial swelling, dissolution and degeneration of lamellar bodies, while vascular ECs displayed hallmark features of necroptosis, such as cell membrane disruption, irregular nuclear chromatin, cytoplasmic leakage and basement membrane discontinuity; in contrast, the air group maintained normal lung structure (Figure 2D). qRT–PCR confirmed that *MLKL* mRNA levels in ECs were significantly elevated at both day 7 and day 14 in hyperoxia‐exposed mice (Figure 2E). Increased *RIPK3* mRNA expression in ECs was also observed at day 14 (Figure S1B). Furthermore, immunofluorescence analysis demonstrated that *MLKL* protein expression progressively increased with prolonged hyperoxia exposure, showing a significant elevation at day 7 and a further increase at day 14 compared with normoxic controls (Figure 2F,G). A similar temporal upregulation of *RIPK3* was also observed (Figure S1C). Western blot analysis further confirmed that 14 days of hyperoxia significantly increas2ed *MLKL* and *RIPK3* protein levels in ECs (Figure 2H,I and Figure S1D,E). Collectively, these re2sults indicate that *MLKL*‐mediated necroptosis is highly activated in this hyperoxia‐induced BPD model, contributing to lung tissue destruction, alveolar simplification and the progression of BPD (Figure 2J).

### 3.3 Inhibition of the *MLKL* Protein in ECs Effectively Alleviated BPD Histological Lesions in Neonatal Mice Exposed to Hyperoxia

**FIGURE 3:**
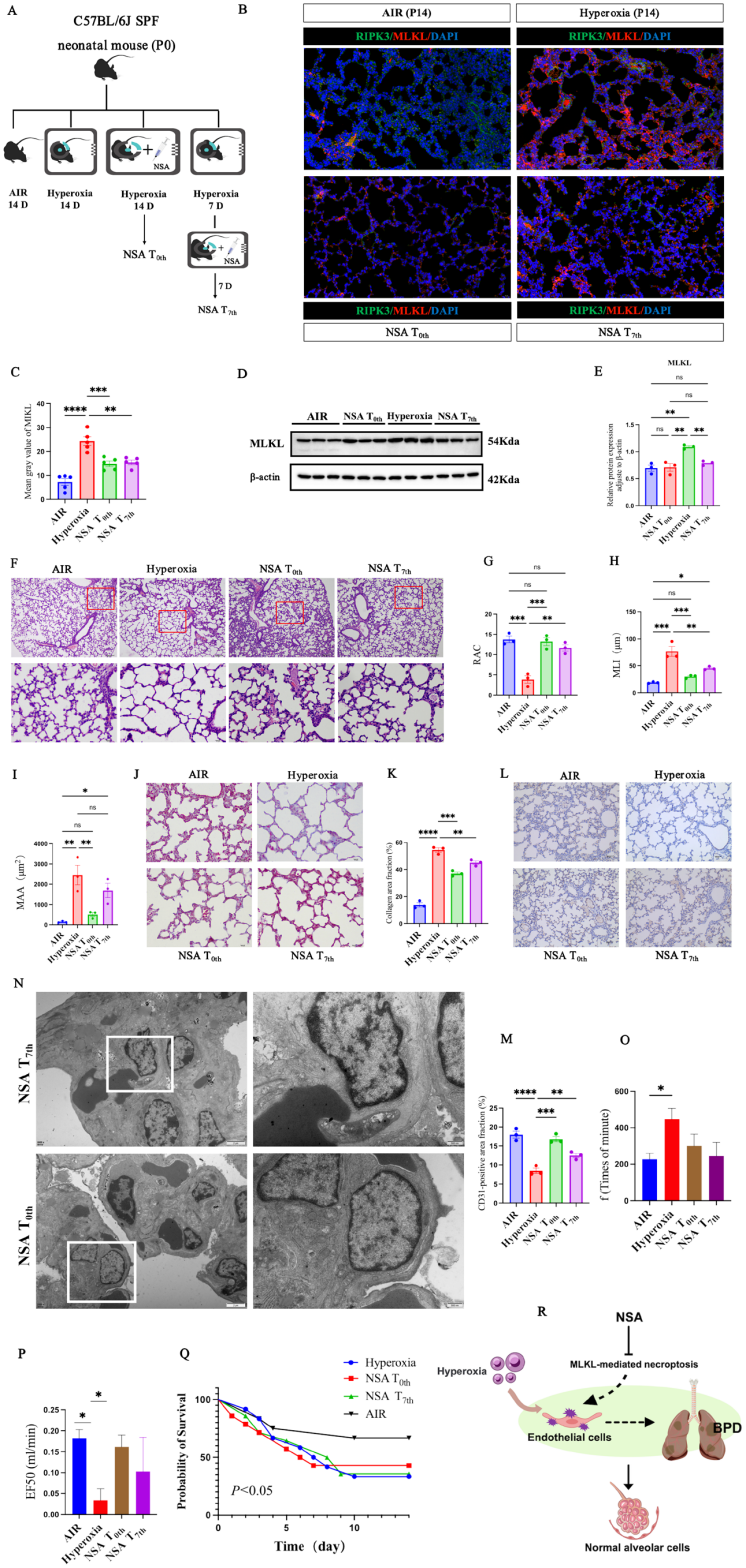
Inhibition of endothelial *MLKL* effectively alleviated histological BPD lesions in neonatal mice exposed to hyperoxia. (A) Endothelial *MLKL* protein expression was inhibited by NSA at two time points: Prior to modelling (NSA T_0th_) and on day 7 after modelling (NSA T_7th_). Respiratory function was measured at P14, and lung tissue was harvested for subsequent analyses; (B) Representative immunofluorescence labelling images (scale bar = 20 μm); (C) Quantification of the data in (B); (D) Western blot analysis of *MLKL* in ECs; (E) Quantification of the data in (D); (F) Representative H&E staining images. The top panels show lower magnification images (scale bar = 100 μm), and the bottom panels show higher magnification images (scale bar = 10 μm); (G–I) Quantification of RAC (G), MLI (H) and MAA (I) based on the data shown in (F); (J) Representative Masson staining images (scale bar = 10 μm); (K) Quantification of the data in (J); (L) Representative CD31 immunohistochemical staining image (scale bar = 40 μm); (M) Quantification of the data in (L); (N) Representative TEM images. The scale bar in the left panel is 2 μm, and that in the right panel is 500 nm; (O–P) Respiratory function analysis; (Q) Survival curves analysis; (R) Schematic illustration demonstrating that inhibiting *MLKL*‐mediated necroptosis preserves EC integrity, maintains alveolar structure and alleviates the pathological features of BPD. * *p* < 0.05, **p < 0.01; ***p < 0.001; ****p < 0.0001, ns > 0.05.

In this study, we administered NSA at two distinct time points (NSA T_0th_ and NSA T_7th_ groups) to assess the impact of endothelial *MLKL* inhibition on BPD development (Figure 3A). Immunofluorescence analysis revealed that *MLKL* protein levels were markedly reduced in the air, NSA T_0th_ and NSA T_7th_ groups compared with the hyperoxia group. In contrast, *RIPK3* protein expression showed no significant differences among the hyperoxia, NSA T_0th_ and NSA T_7th_ groups (Figure 3B,C and Figure S1F). Western blotting confirmed that NSA treatment reduced *MLKL* expression in ECs in both NSA‐treated groups relative to the hyperoxia group (Figure 3D,E). Morphometric analysis of H&E‐stained lung sections demonstrated that, compared with the hyperoxia group, both NSA T_0th_ and NSA T_7th_ treatment significantly increased RAC and decreased MLI. MAA was significantly reduced in the NSA T_0th_ group, whereas the NSA T_7th_ group showed an improving trend in MAA relative to hyperoxia (Figure 3F–I). Quantitative analysis of Masson‐stained lung sections revealed a significantly increased fibrotic area in the hyperoxia group compared with the air group, which was significantly reduced following NSA treatment (Figure 3J,K). Similarly, quantitative analysis of CD31‐immunostained sections showed a significant increase in CD31‐positive area in NSA‐treated groups compared with hyperoxia (Figure 3L,M). TEM revealed that ECs in NSA‐treated groups exhibited less nuclear condensation, preserved plasma membrane integrity and relatively intact ultrastructural organisation compared with the hyperoxia group (Figure 3N). Pulmonary function testing showed that hyperoxia increased respiratory rate, which was partially reduced following NSA treatment. EF50 was significantly lower in the hyperoxia group than in the air and NSA T_0th_ groups, with a modest improvement observed in the NSA T_7th_ group (Figure 3O,P). Survival analysis further showed that NSA administration at both time points was associated with moderately improved survival compared with hyperoxia alone, although survival remained lower than that of air‐exposed controls (Figure 3Q). These findings indicate that inhibiting necroptosis in ECs via NSA, whether as a pretreatment or appropriately delayed treatment, can ameliorate hyperoxia‐induced BPD (Figure 3R).

### 3.4 *MLKL* EC ‐CKO Decreased BPD Lesions in Neonatal Mice

**FIGURE 4:**
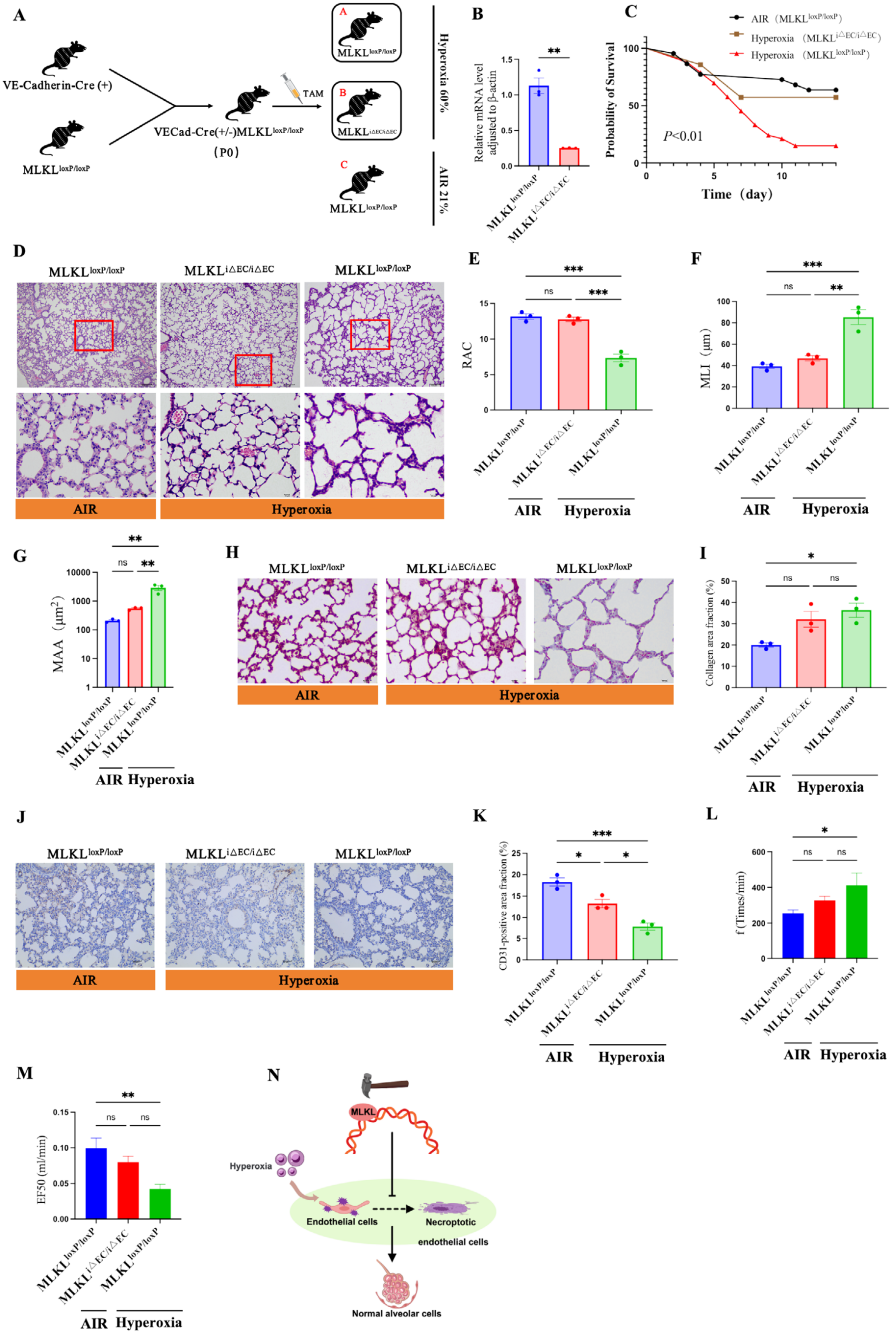
Specific knockout of the *MLKL* gene in ECs decreased BPD lesions in neonatal mice. (A) At P14, the respiratory function of the mice in the EC‐specific *MLKL* knockout (*MLKL* ^i△EC/i△EC^) group and the *MLKL* ^loxP/loxP^ group was evaluated, and lung tissues were collected for further analyses; (B) RT–qPCR analysis of *MLKL* mRNA levels in ECs; (C) Survival curve analysis; (D) Representative H&E staining images. The top panels are low‐magnification images (scale bar = 100 μm), and the bottom panels are high‐magnification images (scale bar = 10 μm); (E–G) Quantitative assessments of the RAC (E), MLI (F) and MAA (G) from the data in (D); (H) Representative Masson staining images (scale bar = 10 μm); (I) Quantitative analysis of the data in (H); (J) Representative CD31 immunohistochemical staining image (scale bar = 40 μm); (K) Quantification of the CD31‐positive area from the data in (J); (L–M) Respiratory function analysis; (N) Schematic illustration demonstrating that EC‐specific knockout of the *MLKL* gene mitigates hyperoxia‐induced BPD. * *p* < 0.05, **p < 0.01; ***p < 0.001; ns > 0.05.

We selectively knocked out the *MLKL* gene in ECs to evaluate its impact on lung pathology in a hyperoxia‐induced BPD (Figure 4A). After 14 days of hyperoxia exposure, RT–qPCR analysis showed that MLKL mRNA levels in ECs were significantly reduced in MLKL^iΔEC/ΔEC^ mice compared with MLKL^loxP/loxP^ controls, confirming efficient gene deletion (Figure 4B). Survival analysis demonstrated that hyperoxia markedly reduced survival in MLKL^loxP/loxP^ mice compared with air‐exposed controls, whereas survival in hyperoxic MLKL^iΔEC/ΔEC^ mice did not differ significantly from that in the air group (Figure 4C). Morphometric analysis of H&E‐stained lung sections showed that RAC, MLI and MAA in hyperoxic MLKL^iΔEC/ΔEC^ mice were significantly improved compared with hyperoxic MLKL^loxP/loxP^ mice and did not differ significantly from air controls (Figure 4D–G). Quantitative analysis of Masson‐stained lung sections further showed that, under hyperoxic conditions, the fibrotic area in MLKL^iΔEC/ΔEC^ mice exhibited a decreasing trend compared with hyperoxic MLKL^loxP/loxP^ mice (Figure 4H,I). In addition, quantitative analysis of CD31 immunostaining revealed a significantly increased CD31‐positive area in the knockout group relative to hyperoxic controls (Figure 4J,K). Pulmonary function testing showed that hyperoxic MLKL^loxP/loxP^ mice displayed an increased respiratory rate and reduced EF50 compared with both MLKL^iΔEC/ΔEC^ and air groups (Figure 4L,M). Collectively, these findings indicate that EC‐specific MLKL knockout significantly protects against hyperoxia‐induced BPD lesions (Figure 4N).

## 4 Discussion

BPD is among the most common complications in preterm infants [32]. Although advancements in oxygen therapy have notably increased the overall survival of these infants, the prevalence of BPD has not shown a corresponding decline; instead, it has shown an increasing trend [33]. As an initial environmental stimulus, oxygen regulates the normal development of the lungs through complex interactions with genes and cells in neonates. However, prolonged exposure to elevated oxygen levels can drive the excessive production of reactive oxygen species, ultimately compromising the lung structure and inducing permanent developmental alterations [34]. In this study, we successfully established an animal model that mimics human BPD by housing neonatal mice in a hyperoxic environment. This model exhibited typical BPD pathological features, including the simplification of alveoli, the dilation of alveolar cavities, an increase in collagen deposition and the obstruction of pulmonary blood vessel growth, which are characteristics similar to those of human BPD patients, thus validating the model. Evaluations of neonatal mice revealed that the EF50 and TV were markedly lower in the hyperoxia group than in the control group, which aligns with the atypical pulmonary function observed in human BPD patients [35, 36]. Additionally, mice subjected to hyperoxia presented significantly lower body weights, indicating that oxygen toxicity may impede growth and development—findings that parallel the growth restrictions reported in children with BPD [37, 38]. Moreover, the survival rate of the hyperoxia‐exposed mice was substantially lower than that of the control mice, reinforcing the deleterious effects of oxygen toxicity on survival, which is consistent with studies in human BPD patients [2, 39]. Compared with 7 days of exposure, 14 days of hyperoxia exposure led to more severe pathological changes in the lungs, especially lung fibrosis and angiogenesis inhibition, indicating that 14 days of exposure more effectively simulated the clinicopathological features of BPD.

Unlike the acute course of ALI, BPD is a chronic disease. Studies have shown that these two diseases have overlapping genetic backgrounds and that both diseases develop under the influence of unfavourable factors such as infection and inflammation. The pathogenesis of BPD involves multiple signalling pathways and multiple genes [40]. Nevertheless, the role of the canonical necroptosis signalling pathway in the pathogenesis of BPD remains incompletely understood. In this study, qRT–PCR analysis revealed a pronounced increase in *MLKL* mRNA expression in isolated pulmonary ECs from neonatal mice exposed to hyperoxia for 14 days. Western blot analysis further confirmed elevated levels of total *MLKL* and p‐*MLKL* in ECs. Consistently, immunofluorescence analysis of whole lung sections demonstrated significantly greater *MLKL* fluorescence intensity in the hyperoxia group compared with controls. TEM further showed that pulmonary vascular ECs from hyperoxia‐exposed mice exhibited ultrastructural features consistent with necroptosis. In addition, the upregulation of *RIPK3* expression in ECs under hyperoxic conditions supports the involvement of upstream necroptosis‐associated signalling. Taken together, these findings suggest that *MLKL*‐mediated necroptosis may contribute to hyperoxia‐induced BPD. We speculate that, as a pivotal downstream component of the necroptotic pathway, *MLKL* becomes activated under hyperoxic conditions and forms pores in the cell membrane, increasing its permeability. This process triggers necroptotic cell death, further amplifying inflammation and structural damage and ultimately hindering normal alveolar development, thereby exerting a persistent negative impact on lung maturation. Previous studies have established that the pharmacological inhibitor NSA effectively suppresses *MLKL*‐mediated necroptosis [27, 28] and has been shown to protect rats against lung ischaemia–reperfusion injury and mitigate Alzheimer's disease progression via *MLKL* inhibition [41, 42]. In the present study, we employed NSA to investigate the role of necroptosis in a validated in vivo BPD model. Western blot analysis and double immunofluorescence labelling indicated that NSA markedly reduced *MLKL* protein expression. Pathological examinations revealed that NSA treatment ameliorated abnormal alveolarisation and obstructed microvascular development in lung tissues relative to those of the control group, and lung function was also correspondingly improved. TEM analysis confirmed that necrotic morphology and ultrastructural damage were substantially alleviated in the mice pretreated with NSA or treated with NSA for 7 days post‐hyperoxia, compared with those in the mice exposed to hyperoxia alone. Overall, these results indicate that *MLKL*‐mediated necroptosis is an important contributor in hyperoxia‐induced BPD in neonates. Specifically, *MLKL*—the terminal executioner of necroptosis—becomes activated under hyperoxic conditions, whereas NSA effectively halts this process by inhibiting *MLKL* activity. This inhibition not only reduces lung injury but also facilitates improved lung tissue remodelling, highlighting *MLKL* as a potential therapeutic target in BPD. Thus, targeting *MLKL* may be promising for normalising lung development and function in patients with BPD.

As survival rates for premature infants have increased, the clinical and pathological features of BPD have likewise evolved. Rather than the severe lung injury originally described by Northway et al. [1], contemporary BPD is predominantly characterised by disordered alveolar development and pulmonary vascular remodelling [43]. These changes reflect a shift in the pathological landscape of BPD. Indeed, one study revealed an abnormal alveolar microvasculature in the lung tissue of infants who died from BPD [12]. In animal models, disrupting vascular endothelial growth factor (VEGF) signalling was shown to hinder alveolar development, culminating in alveolar simplification and diminished pulmonary capillaries; conversely, intratracheal administration of adenovirus‐mediated VEGF gene therapy substantially increased pulmonary capillary formation and preserved normal alveolar architecture [44]. Other studies have shown that administering antiangiogenic agents to neonatal rats impairs pulmonary angiogenesis and leads to alveolar dysplasia [45, 46]. Collectively, these findings suggest that BPD is not solely an air–space disorder and that impaired pulmonary angiogenesis may serve as a key driving factor in BPD pathogenesis. Previous studies have reported that pulmonary vascular ECs are highly vulnerable to hyperoxic stress [13, 14], underscoring their importance in the development of BPD. Accordingly, this study employed *MLKL* ^iΔEC/iΔEC^ neonatal mice under hyperoxia to examine the role of the *MLKL* gene in pulmonary vascular ECs. We observed that the lung pathology and respiratory indices of *MLKL*‐deficient neonatal mice were comparable to those of *MLKL* ^loxP/loxP^ mice under normoxic conditions and were significantly better than those of hyperoxia‐exposed *MLKL* ^loxP/loxP^ mice. These data indicate that the deletion of the *MLKL* gene in ECs has a protective effect on lung structure and function during hyperoxia‐induced injury. Furthermore, our results reinforce the concept that endothelial cell damage may be a key initiating factor of BPD, whereas impaired angiogenesis could be one of the principal mechanisms driving BPD progression.

## 5 Conclusions

In summary, our study demonstrates that hyperoxia disrupts normal alveolar development by activating *MLKL*‐mediated necroptosis in pulmonary ECs, leading to alveolar simplification and structural remodelling characteristic of BPD. Importantly, targeted inhibition of endothelial necroptosis markedly attenuated hyperoxia‐induced lung injury, highlighting endothelial cell dysfunction as a critical contributor to BPD pathogenesis.

## Author Contributions

**Junjie Ning:** investigation, writing – original draft, visualization, formal analysis, project administration, data curation, methodology, writing – review and editing. **Junchao Deng:** investigation, visualization, methodology, formal analysis, software, data curation. **Yating Sang:** investigation, validation, formal analysis. **Lina Qiao:** conceptualization, investigation, funding acquisition, writing – review and editing, formal analysis, project administration, supervision, resources.

## Funding

This study was supported by the Fund of National Key R&D Program of China (2021YFC2701700, 2021YFC2701704).

## Conflicts of Interest

The authors declare no conflicts of interest.

## Supporting information


**Figure S1:** Hyperoxia induces upregulation of *RIPK3* expression in pulmonary ECs of neonatal mice. (A) Volcano plots of the differentially expressed genes in ECs. *p* value < 0.05, |log2FoldChange| ≥ 1; (B) RT–qPCR analysis of *RIPK3* mRNA expression in ECs; (C) Quantification of *RIPK3* protein expression from Figure 2F; (D) Western blot analysis of *RIPK3* in ECs; (E) Quantification of the data in (D); (F) Quantification of *RIPK3* protein expression from Figure 3B. * *p* < 0.05, ** *p* < 0.01; *** *p* < 0.001; ****p < 0.0001, ns > 0.05.

## Data Availability

The data that support the findings of this study are available from the corresponding author upon reasonable request.
