# Supplementary material for: Endothelial MLKL Inhibition Reduces Hyperoxia‐Induced Bronchopulmonary Dysplasia in Neonatal Mice
Source: J Cell Mol Med. 2026 Jan 29;30(3):e71035. doi: 10.1111/jcmm.71035 (PMC12853218; doi:10.1111/jcmm.71035)
Supplement: Supplementary file 1 — Figure S1: Hyperoxia induces upregulation of RIPK3 expression in pulmonary ECs of neonatal mice. (A) Volcano plots of the differentially expressed genes in ECs. p value < 0.05, |log2FoldChange| ≥ 1; (B) RT–qPCR analysis of RIPK3 mRNA expression in ECs; (C) Quantification of RIPK3 protein expression from Figure 2F; (D) Western blot analysis of RIPK3 in ECs; (E) Quantification of the data in (D); (F) Quantification of RIPK3 protein expression from Figure 3B. * p < 0.05, ** p < 0.01; *** p < 0.001; ****p < 0.0001, ns > 0.05. [file JCMM-30-e71035-s001.docx]

**Supplementary Figures**


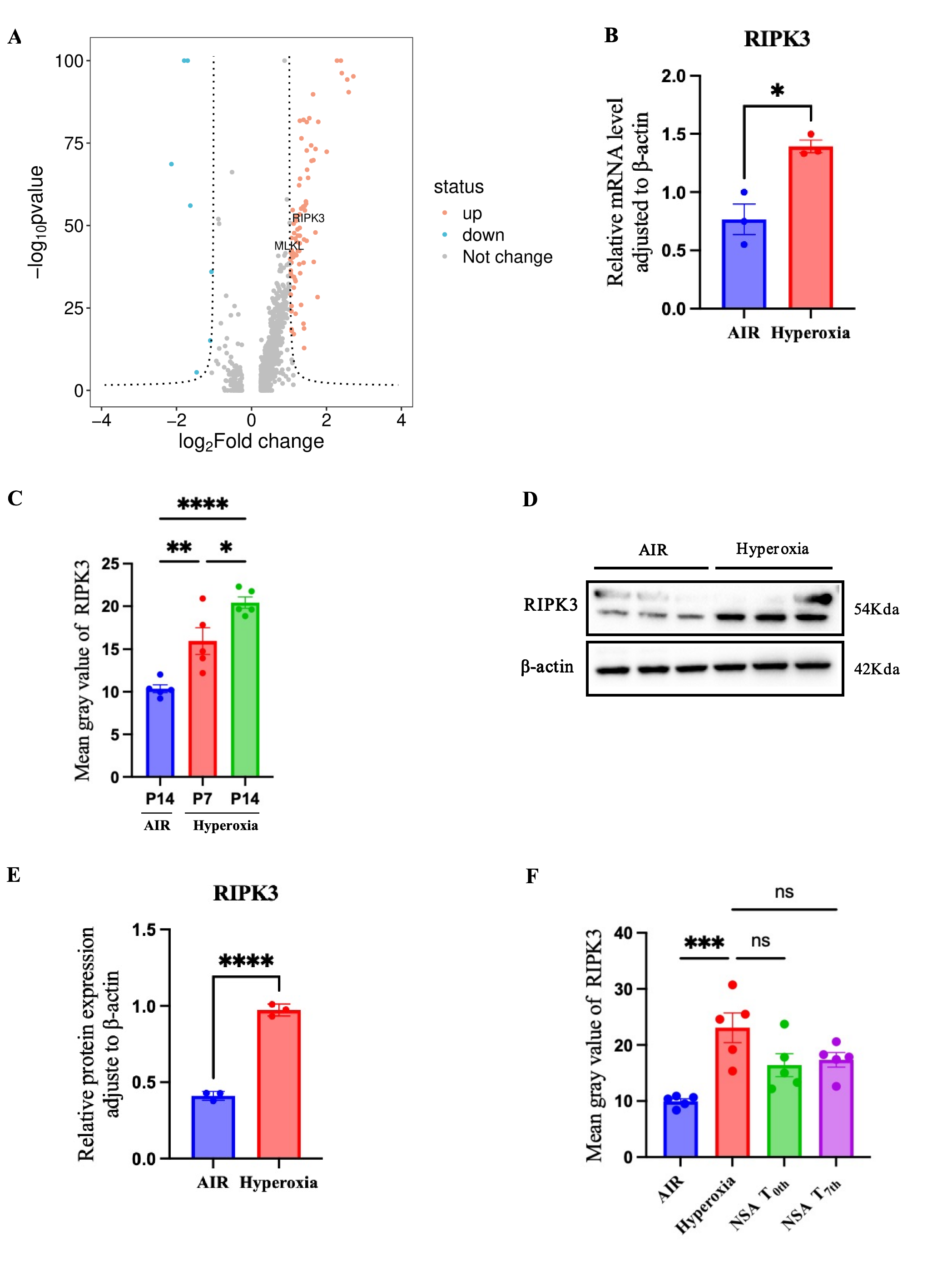


**Figure S1**. Hyperoxia induces upregulation of *RIPK3* expression in pulmonary ECs of neonatal mice. **(A)** Volcano plots of the differentially expressed genes in ECs. p-value < 0.05, |log2FoldChange|≥1; **(B)** RT‒qPCR analysis of *RIPK3* mRNA expression in ECs; **(C)** Quantification of *RIPK3* protein expression from Figure 2F; **(D)** Western blot analysis of *RIPK3* in ECs; **(E)** Quantification of the data in (D); **(F)** Quantification of *RIPK3* protein expression from Figure 3B. * *P* < 0.05, ** *P* < 0.01; *** *P* < 0.001; *****P* <0.0001, *ns* > 0.05.
